# Advice given by community members to pregnant women: a mixed methods study

**DOI:** 10.1186/s12884-016-1146-y

**Published:** 2016-11-09

**Authors:** Bianca A. Verma, Lauren P. Nichols, Melissa A. Plegue, Michelle H. Moniz, Manisha Rai, Tammy Chang

**Affiliations:** 1 Department of Pediatrics, University of North Carolina, 260 MacNider Building CB #7220, 321 S. Columbia Street, Chapel Hill, NC 27599 USA; 2 Department of Family Medicine, University of Michigan, 1018 Fuller Street, Ann Arbor, MI 48104 USA; 3 Department of Obstetrics & Gynecology, University of Michigan, 1500 East Medical Center Drive, Ann Arbor, MI 48109 USA; 4 University of Michigan Medical School, 1301 Catherine Street, Ann Arbor, MI 48109 USA; 5 Institute for Healthcare Policy and Innovation, University of Michigan, 2800 Plymouth Road, North Campus Research Complex, Building 16, Ann Arbor, MI 48109 USA

**Keywords:** Advice, Friends, Family, Pregnancy, Weight gain, Community health, Smoking

## Abstract

**Background:**

Smoking and excess weight gain during pregnancy have been shown to have serious health consequences for both mothers and their infants. Advice from friends and family on these topics influences pregnant women’s behaviors. The purpose of our study was to compare the advice that community members give pregnant women about smoking versus the advice they give about pregnancy weight gain.

**Methods:**

A survey was sent via text messaging to adults in a diverse, low-income primary care clinic in 2015. Respondents were asked what advice (if any) they have given pregnant women about smoking or gestational weight gain and their comfort-level discussing the topics. Descriptive statistics were used to characterize the sample population and to determine response rates. Open-ended responses were analyzed qualitatively using grounded theory analysis with an overall convergent parallel mixed methods design.

**Results:**

Respondents (*n* = 370) were 77 % female, 40 % black, and 25 % reported education of high school or less. More respondents had spoken to pregnant women about smoking (40 %, *n* = 147) than weight gain (20 %, *n* = 73). Among individuals who had not discussed either topic (*n* = 181), more reported discomfort in talking about weight gain (65 %) compared to smoking (34 %; *p* < 0.0001). Advice about smoking during pregnancy (*n* = 148) was frequently negative, recommending abstinence and identifying smoking as harmful for baby and/or mother. Advice about weight gain in pregnancy (*n* = 74) revealed a breadth of messages, from reassurance about all weight gain (“Eat away” or “It’s ok if you are gaining weight”), to specific warnings against excess weight gain (“Too much was dangerous for her and the baby.”).

**Conclusions:**

Many community members give advice to pregnant women. Their advice reveals varied perspectives on the effects of pregnancy weight gain. Compared to a nearly ubiquitous understanding of the harms of smoking during pregnancy, community members demonstrated less awareness of and willingness to discuss the harms of excessive weight gain. Beyond educating pregnant women, community-level interventions may also be important to ensure that the information pregnant women receive supports healthy behaviors and promotes the long-term health of both moms and babies.

## Background

Health behaviors, such as smoking, physical activity, and dietary habits, have a critical impact on obstetric and neonatal outcomes [1]. Smoking in pregnancy is associated with low birth weight, preterm birth stillbirth and placental abruption, while excess weight gain in pregnancy is associated with hypertensive disorders of pregnancy, gestational diabetes, excessive fetal growth, prolonged labor, birth injury, and cesarean delivery [2–5]. Furthermore, excess weight gain in pregnancy may be associated with an increased risk of long-term obesity in both mother and child [6–9].

Pregnant women receive information about weight gain from several sources, including clinicians, the Internet, and family and friends [10, 11]. There is variability in the consistency with which clinicians discuss appropriate weight gain with their pregnant patients: studies report that between 12 and 85 % of pregnant women were counseled correctly by their healthcare provider regarding how much they should gain during pregnancy [12, 13]. Clinicians may be reluctant to discuss the sensitive topic of weight gain with their overweight or obese patients [12, 14, 15]. Additionally, the Internet is a common source of health information among pregnant women with nearly all women (94 %) using the Internet for pregnancy-related information [16–18]. However, one recent study concluded that the most frequently visited webpages on gestational weight gain do not provide accurate and complete information compared to the 2009 Institute of Medicine guidelines for gestational weight gain [19, 20].

Many pregnant women report that the opinions of friends and family influence their weight gain during pregnancy even more than clinicians or the internet [21, 22]. Several models for understanding the effects of interpersonal support demonstrate that information and advice given by family and friends has a unique influence on behavior change because it is within the context of a caring and trusting relationship [23]. Additionally, social supports can facilitate the coping process and mitigate the effects of health stressors, which may explain why women may seek support from friends and family during a pregnancy [24].

Despite the importance of friend and family advice on women’s behavior, few studies have characterized the frequency or content of this advice, and even fewer have directly asked family and friends about their opinions of these behaviors. These studies suggest that the friends and family of low-income, African American pregnant women are likely to encourage “eating for two,” without acknowledging the dangers of too much weight gain, or acknowledging the different recommendations for weight gain depending on pre-pregnancy BMI [11, 25]. One study found that pregnant women believe that weight gain during pregnancy is uniformly vital for having a healthy baby [26].

Health behaviors during pregnancy are influenced by advice from non-medical sources, yet remain poorly characterized in the medical literature [27–31]. The American public’s increasing recognition of the risks of smoking have created social norms that discourage smoking, however similar social norms to discourage excessive gestational weight gain are not well understood [32–34]. The objective of this study was to determine retrospectively whether community members give advice to pregnant women, what advice they give about smoking versus weight gain during pregnancy, and to assess their comfort in speaking about these topics.

## Methods

### Sample

The University of Michigan Institutional Review Board reviewed this study and determined that it was exempt from review as all survey data collected was anonymous. A sample of adults was recruited from a waiting room of an academic primary care clinic in a low-income community. All visitors who were 18 years or older and owned cell phones with text messaging capability were eligible and were asked when entering the waiting room if they were interested in participating. Pregnant females were excluded from analyses given the possibility of social desirability bias. Recruitment was conducted from June through August of 2015.

### Survey instrument and administration

The investigators developed a survey with 51 unique questions (Appendix 1). The survey was distributed via text messaging. Text messaging is used across nearly all age and income groups, and has been shown to be a cost-effective and preferred form of data collection among low-income populations over traditional techniques such as paper, online, and phone surveys [35, 36].

A subset of the survey questions will be described in this study. The survey employed branching logic so that an individual respondent was asked between 17 and 30 questions. The first six questions asked respondents to report their unique survey access code, age, race, education level, phone type, and gender. Survey domains included healthy habits during pregnancy, gestational weight gain, use of preventive care services, and insurance coverage.

Multiple choice and free text questions were asked. Free text questions accepted any answer as valid. Multiple choice questions restricted responses that fell outside the parameters of a valid answer (i.e. answering anything other than yes/no/not sure to “Are you pregnant now?”), which prompted the survey to respond with “Sorry, we do not recognize that answer. Please try again.” Respondents could skip any question by typing the word “skip.”

### Recruitment and data collection

The study staff member asked individuals in the waiting room if they were interested in participating in the study. If the participant responded affirmatively, the recruiter gave him/her a flyer (with the token gift: chapstick or frisbee) and explained how to complete the survey. Participants were encouraged to ask the recruiter any questions or for help with technical difficulties. Participants were able to complete the survey at any location, whether in the waiting room, doctor’s office, or outside the clinic setting. Survey responses were stored and transmitted by *Mosio.com*, a secure online texting platform.

### Analysis

Descriptive statistics were calculated for demographic information and the responses to each of the following close-ended questions: “Have you ever spoken with a pregnant woman about *smoking* during pregnancy?” and ““Have you ever spoken with a pregnant woman about *weight gain* during pregnancy?” McNemar’s test was used to compare the proportion of respondents who had spoken about smoking with those who had spoken about weight gain. Respondents who reported that they have never spoken about these topics before were asked, “Would you feel comfortable talking about this topic with a pregnant woman?” McNemar’s test was also used to compare the proportion that felt comfortable versus those that did not feel comfortable. Logistic regression was used to characterize the groups of individuals who had only spoken about smoking, only spoken about weight gain and those who reported speaking about both.

Respondents who reported speaking to a pregnant woman about either smoking or pregnancy were then asked one of two open-ended questions: “What did you say to her [pregnant woman] about *smoking* during pregnancy?” or “What did you say to her [pregnant woman] about *weight gain* during pregnancy?” respectively. The responses to these open-ended responses were analyzed qualitatively using a grounded theory approach. Two researchers (BV and LN) simultaneously, but independently, identified themes and created preliminary codes. Then through an iterative process to reach consensus, a codebook was created that encompassed both the tone and content of messages. Codes were then applied to responses independently and an iterative process of in-person discussion was used to reach consensus on any discrepancies. The independent strands of qualitative and quantitative data were collected within the same survey and then analyzed independently and combined to create an overall interpretation of the data, consistent with a convergent parallel mixed methods approach. Qualitative data was analyzed using NVivo qualitative data analysis software; QSR International Pty Ltd. Version 10, 2012 [37].

## Results

**Fig. 1:**
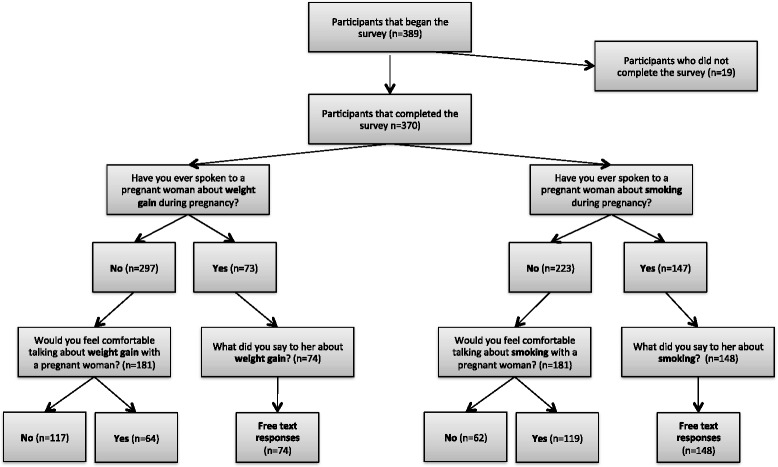
Flow chart of respondents completing survey

A flow chart of how respondents answered questions in the branching logic survey is in Fig. 1.

Invitation fliers were distributed to 1061 adults in the waiting room of the clinic. Of the total sample of non-pregnant females and males over 18 who began the survey (*n* = 389), 370 people completed the survey and responded to both questions about speaking to a pregnant woman about smoking and weight gain during pregnancy (Table 1). Of these respondents, 77 % were female, 40 % were non-Hispanic Black and 25 % reported education of high school or less. The average age was 36 years.

**Table 1:** Demographics of Respondents (*N* = 370^a^)

Variable	
Age–mean (SD)	36.2 (12.8)
Gender—n (%)	
Female	283 (77)
Male	86 (23)
Other	1 (1)
Race/Ethnicity–n (%)	
Non-Hispanic Black	146 (40)
Non-Hispanic White	168 (46)
Hispanic	14 (4)
Asian	14 (4)
Other–Including Mixed	27 (7)
No Answer (Missing)	1
Education–n (%)	
Less than high school	22 (6)
High school graduate	70 (19)
Some college	148 (40)
College graduate or above	128 (35)
No Answer (Missing)	2
Phone type–n (%)	
Smartphone	317 (86)
Flip-Phone	8 (2)
Other	44 (12)
No Answer (Missing)	1

Percentages may not add to 100 due to rounding

^a^Excludes the *n* = 41 respondents who skipped these questions

Over half of respondents in our community-based sample (189/370; 51 %) have spoken to pregnant women regarding either smoking, weight gain in pregnancy, or both. More respondents had spoken to pregnant women about smoking (40 %, *n* = 147) than weight gain (20 %, *n* = 73; *p*-value < 0.001) (Table 2). Sixty percent (223/370) of participants said they had *not* spoken to a pregnant woman about smoking during pregnancy. Among those that had not spoken to a woman about smoking during pregnancy, 34 % said they would feel uncomfortable doing so. In contrast, 80 % of people had *not* spoken to a pregnant woman about weight gain during pregnancy (297/370). Among those who had not spoken to a woman about weight gain during pregnancy, 65 % said they would feel uncomfortable doing so.

**Table 2:** Frequency table of respondents who answered YES or NO to Speaking to Pregnant Women about Smoking or Weight Gain during Pregnancy (*n* = 370)

“Have you ever spoken to a pregnant woman about her smoking during pregnancy”	“Have you ever spoken to a pregnant woman about her weight gain during pregnancy”		
	No	Yes	Total
No	186	37	223 (60 %)
Yes	111	36	147 (40 %)**
Total	297 (80 %)	73 (20 %)**	370

**p < 0.001 using McNemar’s test

Out of the 181 individuals who did not speak to a pregnant woman about either smoking or weight gain, respondents were more uncomfortable talking about weight gain (65 %) compared to smoking (34 %; *p*-value < 0.001) (Table 3).

**Table 3:** Comparing Respondent Comfort Level when Speaking to Pregnant Women about Smoking versus Weight Gain (*N* = 181^a^)

“Would you feel comfortable talking about smoking with a pregnant woman”	“Would you feel comfortable talking about weight gain with a pregnant woman”		
	No	Yes	Total
No	55	7	62 (34 %)**
Yes	62	57	119 (66 %)
Total	117 (65 %)**	64 (35 %)	181

^a^Five respondents did not answer *both* questions about comfort

**p < 0.001 using McNemar’s test

Logistic regression modeling showed that respondents who were female (OR = 4.77, *p*-value = 0.037) and of higher education (OR = 6.21, *p*-value = 0.015) were more likely to speak to pregnant women about both weight gain and smoking (Table 4). Of the 36 individuals who replied affirmatively to both subjects, the majority were higher educated females (*n* = 31, 86.1 %). Both non-Hispanic black (OR = 2.94, *p*-value = 0.017) and “other” race respondents (OR = 4.54, *p*-value = 0.003) were more likely to discuss only weight gain with pregnant women when compared to non-Hispanic white respondents. Respondents in the “other” race group (including mixed, Hispanic and Asian) were less likely to talk to pregnant women only about smoking when compared to non-Hispanic white respondents (OR = 0.43, *p*-value = 0.030; Table 4).

**Table 4:** Logistic Regression of Demographics on Likelihood of Discussing Smoking and Weight Gain

	YES to “Have you ever spoken to a pregnant woman about her smoking during pregnancy” ONLY^a^		YES to “Have you ever spoken to a pregnant woman about her weight gain during pregnancy” ONLY^b^		YES to both previous questions^c^	
	OR (95 % CI)	*p*-value	OR (95 % CI)	*p*-value	OR (95 % CI)	*p*-value
Age in years	0.99 (0.98, 1.02)	0.909	0.99 (0.96, 1.02)	0.471	0.98 (0.96, 1.02)	0.419
Gender^d^						
Female	1.18 (0.67, 2.05)	0.567	0.82 (0.37, 1.83)	0.625	4.77 (1.10, 20.62)	0.037
Male	Reference		Reference		Reference	
Race/Ethnicity						
Non-Hispanic White	Reference		Reference		Reference	
Non-Hispanic Black	0.72 (0.43, 1.20)	0.209	2.94 (1.21, 7.11)	0.017	1.40 (0.64, 3.09)	0.399
Other^e^	0.43 (0.20, 0.92)	0.030	4.54 (1.68, 12.26)	0.003	0.78 (0.21, 2.93)	0.712
Education						
High School or Less	Reference		Reference		Reference	
Some college and Above	0.71 (0.64, 2.05)	0.567	0.82 (0.38, 1.76)	0.611	6.21 (1.44, 26.83)	0.015

^a^Modeling those who said yes to only smoking (*n* = 111) vs. those who said No to discussing smoking (*n* = 223)

^b^Modeling those who said yes to only weight gain (*n* = 37) vs. those who said No to discussing weight gain (*n* = 297)

^c^Modeling those who said yes to both smoking and weight gain (*n* = 36) vs. all others (*n* = 334)

^d^Individual with “other” gender removed from analysis

^e^Includes Asian, Hispanic and Mixed

All participants who responded affirmatively to “Have you ever spoken with a pregnant woman about her smoking during pregnancy?” (*n* = 148) responded to “What did you say to her about smoking?” Qualitative analysis revealed that the most common responses were those that recommended abstinence (“Don’t do it, stop, quit”) (*n* = 59), and identified smoking as harmful for baby (*n* = 97) or mother (*n* = 36) (Table 5). The most common tone was educational or informational (*n* = 83) followed by directive or imperative (*n* = 53), and occasionally the tone was exaggerated or alarming (“Give your child a chance bc the world is already horrible”) (*n* = 10).

**Table 5:** Common themes among people who responded to “What did you say to a pregnant woman about smoking during pregnancy” (*n* = 148)

	Example quotation
Theme	
Don’t do it, stop, quit (*n* = 59)	It’s best to stop Don’t smoke during pregnancy She needed to stop asap
Bad for baby (*N* = 97)	Think about the baby. Give the baby a fair chance at life. Don’t damage the babies [sic] lungs. That it can affect your baby’s health in a negative way. That she should stop because I hear the baby can be smaller or premature That she should stop for her baby and stop being selfish
Bad for mom (*N* = 36)	It’s not good for you or the baby She should not do it during pregnancy she has higher risks of preterm delivery and infections etc.
It might not be that bad (*N* = 15)	Advised her to stop, at least while pregnant and/or nursing. Its okay to smoke but not alot
Asked questions or had conversation about stopping (*N* = 13)	That it’s not good for the baby when is she going to stop Is everything OK? I saw you are pregnant. I thought something must be really wrong if you’re smoking and pregnant Explained the risks it posed, ask if she needed help to quit
Advice about how to stop smoking (*N* = 3)	It may 2 stressful on her child ti quit cild [sic] turkey based 9n her amt she smoked prior 2 her pregnancy “It’s bad for the baby to stop cold turkey because the withdrawal”
You should smoke (*N* = 2)	I was told to take up smoking to lower the birth weight of my child
Tone	
Educating or informational (explaining why) (*N* = 83)	That it was unhealthy for her and the baby I told her it would probably affect the babies development. She didn’t think it would be harmful, but I pointed out that this was the greatest form of second hand smoke
Directive or commanding (*N* = 53)	It’s best to stop Shouldn’t smoke while pregnant
Encouraging or engaging (*N* = 18)	Only encouragement to quit To try to quit smoking, cut down a lot. I suggested quitting smoking.
Blaming or judgmental (*N* = 16)	You shouldn’t smoke during pregnancy it will give your baby birth defects. You want him to have all his toes don’t you? Bad idea. I said “way to go, now your baby can’t breathe fresh air. Yum, I hope she loves second hand smoke”
Non committal (*N* = 11)	It’s your choice Do you smoke more or less?
Exaggerated or alarming (*N* = 10)	Your child will come out with a birth defect Give your child a chance bc the world is already horrible When you smoke your baby smokes

All participants who responded affirmatively to “Have you ever spoken with a pregnant woman about her weight gain during pregnancy?” (*n* = 74) responded to “What did you say to her about weight gain?” The most common messages were reassurance (“It’s ok if you are gaining weight” or “Eat away”) (*n* = 44), warnings against excess weight gain (“Too much was dangerous for her and the baby”) (*n* = 29). Other respondents reported discussions with the pregnant woman about weight gain, how much she had gained, and whether or not she was doing certain health behaviors (*n* = 29) (Table 6). The most common tone was normalizing (*n* = 20), followed by educational or informational (*n* = 18), and very few were blaming or judgmental (*n* = 5).

**Table 6:** Common themes among people who responded to “What did you say to a pregnant woman about weight gain during pregnancy” (*n* = 74)

	Example quotation
Theme	
Normalizing weight gain or reassurance (*N* = 44)	It’s a beautiful thing healthy for the baby I told her it’s OK to gain weight Not to worry about the weight she can lose it That it doesn’t matter how much she gains.
Warnings about weight gain (*N* = 29)	It is important to watch your weight because too much or to less can be harmful to you and the baby Be cautious with to much weight gain to minimize risk of diabetes, but too little can deprive the baby of nutrition. Too much weight gain could lead to gestational diabetes
Conversation or discussion about weight gain (*N* = 20)	Just asked how much did she weigh before her pregnancy and how much did she gain during How much weight have you gain? Do you eat a lot? I asked if her gain was normal and how she felt about it We just discussed it in general. It was a friend who was feeling frustrated about her doctor’s fixation on her weight gain.
Discussed behavior change (*N* = 17)	That’s it’s fine, totally normal, just keep eating healthy and you’ll lose it after the baby is born. Watch what she eat and try to exercise a little bit
Depends on the person or ambivalence (*N* = 8)	That everyone is different some gain more than others they should eat healthy drink lots of water and get some exercise a small to medium amount of weight gain is good in most cases
Told the amount she should gain (*N* = 4)	Should gain 2_3 lbs per monthly Gaining around 30–35 lbs is normal and healthy
Tone	
Normalizing or reassurance (*N* = 20)	Her weight was good You look great! That is was normal and that she shouldn’t worry
Educating or informational (*N* = 18)	Focus on nutrition not cravings WEIGHT GAIN IS GOOD AND BAD DURING PREGNANCY SO EAT HEALTHY!!
Encouraging or engaging (*N* = 18)	It’s a beautiful thing healthy for the baby We talked about what is normal and healthy We just discussed it in general. It was a friend who was feeling frustrated about her doctor’s fixation on her weight gain.
Directive or commanding (*N* = 17)	Don’t gain too much She needed to gain more weight
Non-committal (*N* = 12)	My wife didn’t gain much weight but there wasn’t much concern
Blaming or judgmental (*N* = 5)	You need to stop eating the wrong foods Wow, you are big.

## Discussion

In the setting of increasing rates of obesity among women in the US, there is a particularly pressing need for novel strategies to prevent obesity among women [38]. Our results suggest that respondents in our community-based sample were less comfortable addressing weight gain compared to smoking during pregnancy, a well-documented risk factor for poor maternal and fetal outcomes [2, 3]. This data from community members is consistent with, and adds to, previous studies that demonstrate significant weight stigma and concern for offending pregnant patients among prenatal care providers [12, 14, 15, 39].

Respondents also demonstrated less awareness about the harms of excessive weight gain, and had fewer negatively framed messages for their pregnant peers, compared to smoking. Respondents showed greater understanding of the poor health effects of smoking, had more accurate knowledge about the dangers of smoking (“I asked if she knew the dangers of exposing her unborn child to all the toxins of smoking and the chances of her child having low birth weight or development”), and addressed the issue more directly with pregnant women (“Quitting is the safest and healthiest choice for her and her child”). This reveals that individuals are sharing more varied information about weight gain with pregnant women compared to smoking, suggesting poorer understanding and less comfort in speaking about the harms of excess weight gain compared to smoking.

A potential strategy to prevent the significant morbidity attributable to excess gestational weight gain is to address this common pregnancy risk factor as a public health issue rather than just a clinical issue. Our findings have several important implications. For example, our findings support community-level education (not just to pregnant women) about healthy gestational weight gain as a strategy to change the dialogue and advice surrounding this topic, and increase community members’ comfort in talking about it with pregnant women. Additionally, focusing interventions on sub-groups of people more likely to talk to pregnant women about weight gain, including females with some college education, Non-Hispanic Blacks, and other race groups (Asian, Hispanic, mixed) may prevent the perpetuation of myths.

Responses in our study suggest that friends and family may normalize all weight gain, without discussion of what is *healthy*. By providing community-wide education, through targeted mass media and social media campaigns, it is possible to spread information quickly and effectively about the Institute of Medicine’s guidelines for healthy weight gain during pregnancy [20, 40]. Messages that link recommended weight gain to outcomes that are important to patients (i.e. healthy baby, safe delivery) may improve weight management during pregnancy. Additionally, providing resources and education on the specific types of healthy behaviors that promote healthy weight gain is likely important. Interventions that educate pregnant mothers about healthy behaviors during pregnancy have been shown to result in less weight gain during pregnancy and higher adherence to Institute of Medicine recommendations for gestational weight gain [41]. Community members could be an important source of accurate knowledge for pregnant women to prevent long-term obesity related to pregnancy weight gain.

Participants in our study also demonstrated varied knowledge as to the *specific risks* of excessive weight gain. Educational campaigns clearly highlighting the risks of excess weight gain for mother and baby could help women understand the importance of this issue. Previously studied anti-smoking messaging that emphasizes negative outcomes for the baby has improved quit rates for pregnant women [42, 43].

Further research is needed on the efficacy of widespread public health messaging for healthy pregnancy weight gain and risks of excess weight gain, including how the topics should be framed, and what core messages should be chosen in order to inform women’s weight gain during pregnancy. Intervening during pregnancy has the potential to improve the health of two generations, and to break the cycle of poor health among high-risk populations of women and children [8, 44].

### Clinicians and caregivers may need to fill the knowledge gap

Clinicians and those that provide support and care for pregnant women should acknowledge that friends and family might give inaccurate information about healthy weight gain during pregnancy. As a trusted source of health information, clinicians, nurses, and public health workers should anticipate a knowledge gap about this topic and strive to discuss healthy weight gain with every pregnant woman at every visit [45, 46]. The frequency of prenatal visits allows for revisiting this topic with opportunities to draw on clinical support resources, such as nurses, nutritional counselors, and social workers, to augment the clinician’s efforts. However, these frequent encounters are still less frequent than patients’ encounters with their friends and families, highlighting the importance of addressing this issue outside of the clinical setting. Encouraging friends and family to attend prenatal visits may also help with providing a consistent and personalized message about healthy weight gain to pregnant women.

### Limitations

Our findings are subject to certain limitations. Because we used a convenience sample of respondents from one clinic, we are unable to know the demographics and number of people who chose not to participate. We also did not ask participants their occupation or parental status, which may have influenced their responses. As a result, our findings may be biased towards those with higher incomes, higher educational levels, recent parents, or those who are more comfortable using cell phones to text. Though our sample may not be representative, it does reflects the views of a large group of individuals in the waiting room of a low-income primary care clinic. Of note, enough data was collected to reach thematic saturation in our qualitative analysis of the open-ended responses [47, 48]. While significant, some of the predictors of interest in the regression models provided here did have limited distributions leading to wide confidence intervals for effects on the outcomes. Larger sample sizes would lead to more precise estimates of effects. Validity of data collected via text messaging may differ from traditional forms of data collection. However, studies have shown text messaging surveys to be as valid as paper surveys, and that respondents are more likely to be honest and provide higher quality data in text message surveys compared to verbal interviews [49, 50]. Finally, our research did not explore the limitations experienced by community members in speaking about weight gain during pregnancy, or the accuracy of the advice they provided. These would be potential next steps for research, and further our understanding of the role of community members in promoting healthy weight gain during pregnancy.

## Conclusions

Friends and family are important sources of information in pregnant women’s lives. Our study suggests that community members give pregnant women advice during pregnancy, though may be less knowledgeable and comfortable about the harms of excessive weight gain compared to smoking. Though the harms of smoking during pregnancy are clear to community members, the harms of too much weight gain may not be clear. Beyond educating pregnant women, community-level interventions may also be important to ensure that the information pregnant women receive supports healthy behaviors and promotes the long-term health of both mothers and babies.

## Appendix 1

### Text messaging survey questions

NOTE: Questions not analyzed in this paper are not presented below.

1. Thank you for taking this survey! It should only take a few minutes. To skip a question, type “skip”. To begin, enter the 4 digit code from your info sheet.
2. We’d like to know a little bit about you. What is your age?
3. What is your race/ethnicity?
  - Non-Hispanic BLACK
  - Non-Hispanic WHITE
  - HISPANIC
  - AISAN
  - OTHER race–including Mixed Race
4. What type of phone are you using?
  - Smartphone like Apple iPhone or Samsung Galaxy
  - Flip-phone
  - Other
5. What is the highest level of school you have completed?
  - Less than High School
  - High School Graduate
  - Some College
  - College Graduate or above
6. What is your gender?
  - Male
  - Female
  - Other
7. Are you pregnant now?
  - Yes
  - No
  - Not sure
8. Thanks! The next few questions are about pregnancy. It doesn’t matter if you are male or female. We just want to know your opinion. In your opinion, what is the most important thing a woman should do during pregnancy to have a healthy pregnancy? Please be specific.
9. Have you ever spoken with a pregnant woman about her SMOKING during pregnancy?
  - Yes
  - No
10. What did you say to her about smoking? [Free Text Response]
11. Would you feel comfortable talking about this topic with a pregnant woman?
  - Yes
  - No
12. Have you ever spoken with a pregnant woman about her WEIGHT GAIN during pregnancy?
  - Yes
  - No
13. What did you say to her about weight gain during pregnancy? [Free Text Response]
14. Would you feel comfortable talking about this topic with a pregnant woman?
  - Yes
  - No
